# Myasthenia Gravis: Epidemiology, Pathophysiology and Clinical Manifestations

**DOI:** 10.3390/jcm10112235

**Published:** 2021-05-21

**Authors:** Laura Dresser, Richard Wlodarski, Kourosh Rezania, Betty Soliven

**Affiliations:** Department of Neurology, University of Chicago, Chicago, IL 60637, USA; richard.wlodarski@uchospitals.edu (R.W.); krezania@neurology.bsd.uchicago.edu (K.R.); bsoliven@neurology.bsd.uchicago.edu (B.S.)

**Keywords:** myasthenia gravis, acetylcholine receptor, autoantibodies, cytokines, B cells, T cells

## Abstract

Myasthenia gravis (MG) is an autoimmune neurological disorder characterized by defective transmission at the neuromuscular junction. The incidence of the disease is 4.1 to 30 cases per million person-years, and the prevalence rate ranges from 150 to 200 cases per million. MG is considered a classic example of antibody-mediated autoimmune disease. Most patients with MG have autoantibodies against the acetylcholine receptors (AChRs). Less commonly identified autoantibodies include those targeted to muscle-specific kinase (MuSK), low-density lipoprotein receptor-related protein 4 (Lrp4), and agrin. These autoantibodies disrupt cholinergic transmission between nerve terminals and muscle fibers by causing downregulation, destruction, functional blocking of AChRs, or disrupting the clustering of AChRs in the postsynaptic membrane. The core clinical manifestation of MG is fatigable muscle weakness, which may affect ocular, bulbar, respiratory and limb muscles. Clinical manifestations vary according to the type of autoantibody, and whether a thymoma is present.

## 1. Introduction

Myasthenia gravis (MG) is the most common autoimmune disorder that affects the neuromuscular junction. MG is largely a treatable disease but can result in significant morbidity and even mortality. This can usually be prevented with a timely diagnosis and appropriate treatment of the disease. MG is a heterogeneous disease from a phenotypic and pathogenesis standpoint. The spectrum of symptoms ranges from a purely ocular form to severe weakness of the limb, bulbar and respiratory muscles. The age of onset is variable from childhood to late adulthood with disease peaks in younger adult women and older men [1].

MG is considered a classic example of antibody-mediated autoimmune disease. It can also be viewed as an example of a class II hypersensitivity reaction, as IgG autoantibodies react with intra or extracellular antigens, leading to end-organ damage. Most patients with MG have autoantibodies against the acetylcholine receptors (AChRs) [2,3], and a minority are seropositive for antibodies directed to muscle-specific kinase (MuSK) [4,5], low-density lipoprotein receptor-related protein 4 (Lrp4) [6,7] or agrin [8,9]. These antibodies also provide the basis for defining disease subgroups and help delineate phenotypic variants. In a subgroup of MG patients, striational antibodies have also been identified, which include antibodies against titin, ryanodine receptor, and the alpha subunit of the voltage-gated K+ channel (Kv1.4). These antibodies mostly serve as biomarkers of disease severity and are often detected in patients with late-onset MG or with thymoma, and some of them have concomitant myositis and/or myocarditis [10,11].

Although MG is mediated by autoantibodies, different subtypes of T cells and their cytokines also play important roles in the pathogenesis. In this review, we briefly discuss the epidemiology, clinical manifestations, and genetic predisposing factors of adult MG, then provide an overview of pediatric MG, followed by an update on MG pathophysiology.

## 2. Epidemiology

MG is a rare neurological disease and pediatric MG is even more uncommon. Both incidence and prevalence have significant geographical variations, but it is believed that MG incidence has increased worldwide over the past seven decades. The prevalence of MG was estimated at 1 in 200,000 from 1915 to 1934, increased to 1 per 20,000 after the introduction of anticholinesterase drugs in 1934, and rose to 1 per 17,000 population after the discovery of AChR antibodies in 1969 [12]. Prevalence rates range from 150 to 200 cases per million, and they have steadily increased over the past 50 years, at least partly due to improvements in recognition, diagnosis, treatment, and an overall increase in life expectancy [13]. More recent studies addressing incidence rates have been conducted in Europe and show a wide range from 4.1 to 30 cases per million person-years [14,15].

The annual rate is lower in studies coming from North America and Japan, with the incidence ranging from 3 to 9.1 cases per million [1]. Lower incidence and prevalence rates have been reported in a large study from China at 0.155–0.366 per million, and 2.19–11.07 per 100,000, respectively [16]. Two population-based studies from Korea showed a prevalence of 9.67–10.42 per 100,000 people in 2010, which increased to 12.99 per 100,000 in 2014 [17,18]. On the other hand, a smaller study using records of a hospital-based Health Maintenance Organization estimated an incidence of MG at 38.8 per 1,000,000 person-years for the Argentinian population [19]. Different study methodologies, including diagnostic criteria and other sources of bias, such as the small size of the study population and the underestimation of patients with milder disease, likely play a factor in the significant variability of incidence rates over time and across different geographical regions.

Incidence rates have a bimodal distribution in women, with peaks around age 30 and 50. In men, the incidence increases steadily with age and with the highest rates between age 60 and 89 [20]. Women are more commonly affected before age 40, with a female: male ratio of 3:1 for early-onset MG. In the fifth decade of life, women and men are equally affected, while men have a higher proportion after age 50, with a male: female ratio of 3:2 [21]. Around 10% of cases are pediatric, which is defined as onset before age 18 [13]. MG can affect people of all race and ethnic backgrounds and is slightly more prevalent in patients of African ancestry [22,23,24]. Furthermore, MG phenotype may vary depending on the ethnic background. In a retrospective study from South Africa, black patients were more likely to have treatment-resistant ophthalmoplegia and ptosis than whites, whereas the whites were more likely to develop treatment refractory generalized MG [25]. The age at diagnosis was 17 years higher in Caucasians than non-Caucasians in another cohort of patients with ocular MG [26]. In a US study, Oh et al. found that MG started earlier and had a more severe phenotype in African Americans than in Caucasians [22]. The seronegative African Americans had a higher percent of MuSK seropositivity in that study (50% vs. 17% in the whites). On the other hand, patients of Asian ancestry have higher rates of MuSK antibodies compared to Caucasians and individuals of African ancestry [27,28]. MuSK-associated MG is also more prevalent among those living in latitudes closer to the equator [22,29].

The mortality rate of MG has dramatically declined from the early 20th century after the availability of acetylcholine esterase inhibitors, immunosuppressants, intravenous immunoglobulin and advanced respiratory care. However, the mortality rate from the disease remains at 5–9%, being slightly higher in males than females [12]. Using the US Nationwide Inpatient Sample (NIS) database for the years 2000 to 2005, the overall in-hospital mortality rate was estimated as 2.2%, but higher in those with MG crisis (4.7%), with the main predictors of death being older age and the presence of respiratory failure [24].

## 3. Subtypes of MG and Their Clinical Manifestations

### 3.1. MG Due to Antibodies against AChR (AChR-MG)

#### 3.1.1. Effector Mechanisms

Nicotinic AChR is a heteropentamer consisting of two α-subunits and one each of β-, δ-subunit, and γ-subunit (embryonic type) or ε-subunit (adult type), which are organized around a central pore [30]. Antibodies against the AChR are found in approximately 80% of MG patients. At least half of the AChR autoantibodies are directed at the AChR α-subunits [31]. They are believed to be more pathogenic than those directed against the beta subunit [32]. This is likely due to the location of the alpha subunit within the receptor, which leaves it more exposed to antibodies, as well as its role in modulating the receptor sensitivity to ACh binding [33,34]. In addition, there are two alpha subunits per receptor.

AChR antibodies are predominantly of the IgG1 and IgG3 subclasses [35]. IgG2 and IgG4 subclasses are also identified, but in fewer cases [36]. The pathogenic mechanisms and functional spectrum of AChR antibodies are varied, but overall, they impair receptor function by either binding, blocking, or modulating its activity. The predominant mechanism is the binding of the antibody and activation of the complement cascade, leading to the formation of the membrane attack complex (MAC), which causes damage of the postsynaptic membrane and destruction of synaptic folds which contain AChRs and associated proteins, including voltage-gated sodium channels [35]. Other mechanisms of pathogenicity include: (1) antigenic modulation by the binding and crosslinking of AChRs, leading to increased endocytosis and degradation [37]; and (2) the impairment of AChR function, either by the blocking of ACh binding to the receptor [38] or the prevention of channel opening [39]. Most blocking antibodies appear in association with binding antibodies, and only rarely are they unique. Therefore, this final mechanism is believed to be rare and its clinical implications are not clear [40]. However, the administration of blocking antibodies causes an acute and severe weakness in rodents [41].

#### 3.1.2. Clinical Manifestations

The core clinical manifestation of MG is fatigable muscle weakness, worsened by exertion and improved by rest. The most common presenting symptoms are ocular, with double vision and ptosis. Most patients will develop diplopia and/or ptosis some time during the course of their disease. In addition, up to 80% of patients with ocular onset will go on to develop generalized symptoms, usually within two years of disease onset [42,43]. A recent population-based study conducted by the Mayo Clinic found that 51% of patients presented with ocular onset, and 55% of these developed generalized symptoms [44]. Bulbar muscles are also frequently involved, resulting in flaccid dysarthria, dysphagia, and facial and jaw weakness [45]. Axial weakness can also be present, with neck flexion weakness usually more common than weakness of neck extension. In a previous retrospective study from our center, about 10% of MG patients developed head-drop some time during the disease course [46]. Head-drop was associated with age >60 and male patients in that study. Limb muscle weakness tends to be symmetric and proximal, and patients commonly complain of difficulty climbing stairs, getting up from chairs, and raising arms above their head. In some instances, distal muscles can be predominantly affected, either in a symmetric or asymmetric distribution. For example, some patients complain of weakness in finger and wrist extension and flexion, as well as foot drop. Finally, 15–20% of patients with AChR-MG can develop respiratory weakness requiring mechanical ventilation (MG crisis) [21]. Spontaneous remissions for different lengths of time may occur in the course of adult-onset MG. In an earlier study conducted before the widespread use of steroids and other immunosuppressants, approximately one fourth of the patients had a complete or near complete spontaneous remission, lasting an average of 4.6 years and up to 17 years [47]. Half of the remissions occurred during the first year after onset. A study of Oosterhuis et al. found that 22% of patients treated with anticholinesterase medications only had spontaneous remission [42]. The remission lasted more than 12 months in duration in half of those patients, with the maximum duration of the remission being 6 years in that study.

#### 3.1.3. AChR MG Subtypes

##### Ocular MG

Most MG patients with ocular symptoms at onset will progress to generalized forms of the disease, usually within two years of onset. Of the remaining, 90% will continue to have ocular manifestations only. Hence, ocular MG is defined by isolated extra-ocular involvement for a period of ≥2 years. Over half of the patients in this group have antibodies against AChRs [21]. There are several explanations as to why extraocular muscles (EOMs) are preferentially affected in MG. EOMs fatigue easily, as they require tonic contractions to sustain gaze in a specific direction, and fibers have a high frequency of synaptic firing, and develop tension faster [48]. In addition, EOMs have a lower density of AChR, thus making them more susceptible to symptoms. It is also theorized that differing epitope expression in EOMs plays a role in their preferential involvement [49].

##### Generalized AChR Ab Positive MG (AChR-MG): Early vs. Late Onset

Early-onset MG (EOMG) corresponds to patients presenting before age 50. There is a female preponderance, with a female to male ratio of 3:1. Patients in this category have a higher incidence of thymic hyperplasia, and thymectomy has been proven effective in improving clinical outcomes and minimizing the need for immunotherapy [50]. Late-onset MG (LOMG) is defined by onset after the age of 50. There is no female predominance in this group; on the contrary, there can be a slightly higher prevalence among men, especially after age 60. Thymic hyperplasia is rare and response to thymectomy is poorer [45].

There is a higher prevalence of autoimmune disease among family members and a high correlation with HLA-DR and HLA-B8 haplotypes [51]. There is frequent association of A1-B8-DR3 haplotype with early-onset MG [52]. In a study on non-thymomatous AChR Ab + LOMG of Italian ancestry, Spagni et al. found a positive association with HLA-DRB1*07 and HLA-DQB1*02, whereas HLA-DRB1*02, HLA-DRB1*03, HLA-DRB1*11, and HLA-DQB1*03 were the protective alleles [53]. A study comparing cohorts of patients with EOMG, LOMG and MuSK-MG in a single center in Turkey found a strong association for class I HLA-B/MICA in EOMG patients, specifically HLA-B*08:01. On the other hand, no association was found between LOMG and HLA class I, but it detected an association with HLA-DQA1 and HLA-DRB1 [54]. Another large study on Norwegian MG patients older than 60 showed a strong association with HLA-DRB1*15:01 [55].

Genetic variations within other loci have been associated with predisposition to AChR-MG. A large genome-wide association study involving patients from North America and Italy identified different haplotypes across the HLA region, cytotoxic T-lymphocyte-associated protein 4 gene (CTLA4) and tumor necrosis factor receptor 4 superfamily, member 11a, (TNFRSF11A) and NFκB activator genes in early- and late-onset MG cases [56]. Variations in the CTLA4 and HLA-DQA1 loci were associated with both early- and late-onset cases, whereas genetic variation within the TNFSRF11A locus was a susceptibility factor only in the late-onset cases in that study.

##### Thymoma-Associated MG

MG is the most common paraneoplastic disorder associated with thymoma. Other thymoma-related disorders with lower association rates include myositis, Morvan syndrome and pure red aplasia. About 50% of patients with a thymoma develop positive AChR antibodies without clinical manifestations, and approximately 30% will develop MG [57]. Conversely, 10–20% of patients with MG have thymomas [58]. Response to thymectomy is variable, usually worse than in patients with EOMG [50]. Studies on HLA alleles did not reveal a consistent association between HLA and thymomatous MG [59].

### 3.2. MuSK Antibody-Associated MG (MuSK-MG)

#### 3.2.1. Effector Mechanisms

**Figure 1:**
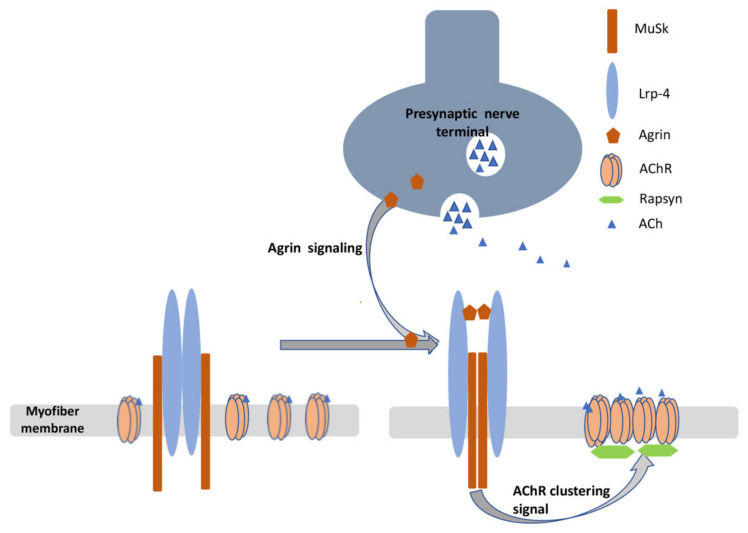
Diagram depicting the secretion of agrin from the presynaptic membrane and its interaction with Lrp4, which results in reorganization and reorientation of MuSK, promoting a signaling pathway that leads to synaptic differentiation, including clustering of AChRs. This involves recruiting rapsyn which links AChRs to the cytoskeleton (not shown).

MuSK is a membrane protein that is critical to the clustering of AChRs in the neuromuscular junction. Agrin, which is secreted from the presynaptic terminal, interacts with Lrp4, resulting in the reorientation of the Lrp4/MuSK complex, which in turn leads to the activation of MuSK through its phosphorylation. Phosphorylated MuSK activates a downstream signaling pathway that leads to the clustering of AChRs (Figure 1) [60].

Antibodies against MuSK are found in approximately 7–10% of all MG patients and up to 40% of patients with generalized MG who are seronegative for AChR Abs. There is a female predominance, with up to 85% of MuSK positive patients being female [61]. Animal studies demonstrated that MuSK antibodies are pathogenic, as they cause severe weakness when administered to healthy mice [62]. In contrast to AChR antibodies, MuSK antibody titers correlate with disease severity [63]. The concurrence of seropositivity for both AChR and MuSK antibodies has rarely been reported [64], but in general these are considered distinct entities.

MuSK antibodies belong mainly to the IgG4 subclass. They do not fix complement and are not strong activators of cell-mediated cytotoxicity [65]. Given the unique ability of IgG4 to undergo Fab arm exchange, MuSK antibodies are functionally monovalent, and they cannot crosslink antigens of the same class. The mechanism by which MuSK antibodies exert their pathogenic effect on the neuromuscular junction is via binding to the Ig-like domain of the protein, preventing its phosphorylation, and subsequently disrupting the Agrin-Lrp4-MuSK-Dok-7 signaling pathway. Dok-7 is a muscle-intrinsic activator of MuSK and it is required for synaptogenesis [66]. This ultimately causes a reduction in the density of AChRs in the postsynaptic membrane [67,68].

#### 3.2.2. Clinical Manifestations

MuSK-MG predominantly affects young adults, and is more prevalent among patients of African descent and those living close to the equator in European and Asian nations [45]. This is likely due to a genetic predisposition and not to environmental factors. Muscle weakness preferentially affects cranial and bulbar muscles. Over 40% of patients present with bulbar weakness, usually associated with neck and respiratory involvement [69,70]. Some patients have tongue atrophy. About 30% of patients present with diplopia and/or ptosis. Limb weakness can be uncommon, but when present it tends to be severe and associated with muscle atrophy. Diurnal variations in strength are less common. There is no clear association between MuSK-associated MG and thymic pathology [21]. From the genetic predisposition standpoint, MuSK-associated MG has been associated with DQB1*05 and HLA-DRB1*14/DRB1*16 [71,72].

Patients who are seropositive for both AChR and MuSK are rare, and it is not certain if they should be categorized as an MG subtype. In a study from southern China, Zhang et al. demonstrated that the phenotype of double-seropositive patients is more severe than AChR-MG and more similar to the MuSK-associated MG [73].

### 3.3. Double-Seronegative Generalized MG

This is a heterogenous group of patients who share negative results for AChR and MuSK antibody testing. Cell-based assays can detect lower titers of these antibodies in patients previously reported as double-negative by more common serologic assays. It is likely that this subgroup of patients has antibodies against antigens not yet identified [51]. In general, these patients present similar to those positive for AChR antibodies in regard to the distribution of muscle weakness, severity, and response to treatment. Cortes-Vicente and colleagues reported a cohort of double-seronegative MG patients who presented mainly with milder forms of disease at onset, less bulbar involvement, and younger age at presentation when compared to AChR-MG [74].

### 3.4. Lrp4 Antibody-Associated MG (Lrp4-MG)

#### 3.4.1. Effector Mechanisms

Lrp4 is the postsynaptic receptor of nerve-derived agrin. The binding of agrin to Lrp4 activates MuSK and initiates a cascade of events leading to the aggregation of AChRs in the neuromuscular junction. Lrp4 antibodies are present in 2–50% of double seronegative MG cases [6,75,76]. Many Lrp4-MG patients also have antibodies against agrin. Lrp4 antibodies are mostly of the IgG1/IgG2 subclass and are believed to be directly pathogenic by disrupting the activation of MuSK [75]. In patients with congenital myasthenic syndromes due to gene mutations in agrin, neuromuscular junctions are less stable and AChRs are more dispersed and un-clustered. Given these findings, the most likely pathologic mechanism of anti-Lrp4 antibodies is a reduction in AChR clustering. Other mechanisms are possible as well, as IgG1 can activate complement [6,76].

#### 3.4.2. Clinical Manifestations

There is a female predominance, and age at onset is variable, but patients tend to present before age 50. Patients can present with ocular or generalized MG, but it is believed that symptom severity is overall milder in this subgroup. About 20% have only ocular symptoms after 2 years of disease [75]. However, patients with a combination of anti-Lrp4 and anti-agrin antibodies may have more severe symptoms [75]. Of note, Lrp4 Ab has also been reported in other neurological diseases, including amyotrophic lateral sclerosis [77,78]; thus, further studies are required to validate its specificity in the diagnosis of MG. The predisposing genetic markers for Lrp4 and double-seronegative MG have not been reported.

## 4. Pediatric MG

Juvenile MG (JMG) is defined as MG in patients younger than 18 years of age. A meta-analysis of epidemiological studies estimated the incidence of JMG between 1 and 5 cases per million person-years [1]. JMG is, however, more prevalent in the Asian than the European populations. For example, Taiwanese and Japanese studies showed incidence rates ranging from 3.7 to 8.9 per million person-years [1,79]. In other studies on the Asian population, JMG constituted up to 50% of total MG cases, mostly of ocular type [80,81]. There is also a higher prevalence in patients of African descent compared to Caucasians [82,83]. Pediatric cases constituted 10–15% of total MG cases in a US study [13]. Similar to the young adults, JMG has a female preponderance [84,85].

A total of 16–38% of JMG cases are ocular [83,84]. Ocular JMG is more common in pre-pubertal children, and post-pubertal children tend to have a greater proportion of generalized MG [84,85,86,87]. As ocular symptoms often occur during critical times during a child’s development, there is a risk for long-term sequelae such as strabismus and amblyopia if this condition is not treated aggressively [88]. The generalization of symptoms occurs in 20–25% of JMG patients [83,88], much lower than the rate of generalization in adults, which can be up to 80% [12]. The course of generalized JMG is unpredictable and may be associated with recurrent exacerbations including myasthenic crisis. On the other hand, JMG patients may undergo periodic spontaneous remissions, at a higher rate than the adults, often lasting for years [83,89].

Similar to adult-onset MG, the most common pathogenic antibodies detected in JMG are AChR Ab, followed by MuSK and Lrp4. Fifty-three to 86% of generalized and ~40% of ocular JMG patients are seropositive for AChR Abs [83,84,90]. It should be noted that ~40% of patients seronegative for AChR Ab may become seropositive within 2 years of follow up [89]. Up to 40% of JMG patients who are seronegative for AChR Ab are seropositive for MuSK Abs [83,91]. Similar to adult-onset MG, anti-MuSK Ab seropositivity is predictive of a more severe phenotype often associated with respiratory and bulbar muscle weakness and atrophy. JMG may also be associated with anti-Lrp4 Ab, which manifests with a milder, predominantly ocular phenotype [91,92]. In a large retrospective study from China, 13 of 455 (2.9%) patients who were seronegative for AChR and MuSK Abs had Lrp4 antibodies; 53.8% of them were children [92]. The pathogenesis of JMG is similar to the adult cases and is described above.

JMG is a separate entity from transient neonatal MG, which occurs in 10–15% of neonates born to mothers with MG as a result of the passive transfer of maternal antibodies in utero [93,94]. While neonatal MG usually resolves spontaneously over weeks to months, the affected infants may have generalized hypotonia, respiratory distress, poor suck in addition to extraocular muscle weakness, necessitating the use of respiratory support, treatment with neostigmine and, in severe cases, plasma exchange [93,94]. Furthermore, the transfer of antibodies directed to fetal AChRs may result in persistent myopathic features (i.e., fetal AChR inactivation syndrome), with symptoms ranging from mild facial and bulbar weakness in the affected infants to arthrogryposis multiplex congenita [95]. Other JMG mimics include congenital myasthenic syndrome, mitochondrial diseases, demyelinating polyneuropathies, and congenital myopathies. The asymmetric nature of ptosis and ophthalmoparesis in ocular JMG may help distinguish it from congenital myasthenic syndromes and mitochondrial diseases, as the extraocular weakness and ptosis in the latter are usually, but not always, symmetrical [96]. Congenital myasthenic syndromes are a particularly heterogeneous group of disorders and are beyond the scope of this chapter.

## 5. Pathophysiology

### 5.1. Physiology and Organization of the Neuromuscular Junction

**Figure 2:**
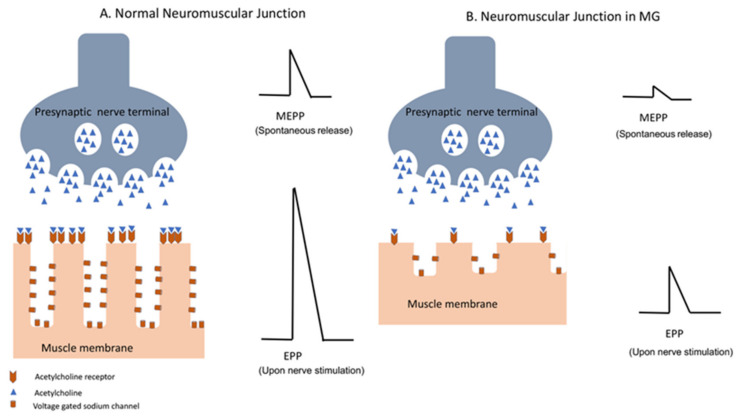
Neuromuscular transmission in normal individuals (**A**) and in patients with MG (**B**). Decreased density of the AChR and complement-mediated damage to the postsynaptic membrane in MG patients result in decrease in miniature end plate potential (MEPP), which occurs with spontaneous release of AChR vesicles, as well as endplate potential (EPP) in response to nerve action potential of the presynaptic membrane. Diminished amplitude of EPP in MG results in impaired neuromuscular transmission.

The neuromuscular junction is the site of impulse transmission between nerve terminals and muscle fibers. This process requires the release of presynaptic acetylcholine (ACh) and its subsequent binding to a postsynaptic ACh receptor. Synaptic vesicles containing ACh are released from the presynaptic membrane after an action potential activates voltage-gated calcium channels, allowing an influx of calcium into the nerve terminal [97]. The diffusion time of ACh through the synaptic cleft is very short, and it is modulated by the enzyme ACh esterase (AChE), which promotes ACh degradation. The spontaneous release of synaptic vesicles generates the so-called miniature end plate potential (MEPP), while upon nerve fiber stimulation/depolarization, a synchronous release of many synaptic vesicles causes large depolarization of the end plate membrane, generating an evoked endplate potential (EPP) (Figure 2). This, in turn, triggers an action potential in the myofiber, which ultimately leads to its contraction. The amount of ACh released into the synapse is usually higher than that required to generate an action potential, which allows for reliable transmission [97,98]. The binding of ACh to its receptors in the postsynaptic membrane opens the ACh cation-specific channel, leading to localized depolarization and activation of adjacent voltage-gated sodium channels. This allows for the translation of the chemical reaction into an electric signal, which is the muscle fiber action potential. The role of AChE in the hydrolyzation of ACh is therefore crucial, as it prevents a single molecule of ACh from repetitively activating AChRs. The effectiveness of neuromuscular junction transmission is also determined by the amount of ACh released by the nerve terminal, the density of ACh receptors in the postsynaptic membrane, and the density of voltage-gated sodium channels at the endplate. The latter is dependent on the presence of folds in the postsynaptic membrane. These folds determine the density of the voltage-gated sodium channels in the postsynaptic membrane, and therefore increase the efficient coupling of the localized EPP to the myofiber action potential [98].

Neuromuscular junction disorders such as MG disrupt the cascade of events that lead to reliable muscle contraction. In addition, there is a reduction in the number of AChRs and voltage-gated sodium channels as the result of complement-related injury to the postsynaptic membrane in MG. The resultant inefficient neuromuscular transmission is reflected in decremental response in the amplitude of compound muscle action potential (CMAP) during repetitive nerve stimulation (RNS) and abnormal jitter or blocking in single fiber EMG [98,99].

### 5.2. Immune Dysregulation in MG

#### Defective B Cell Tolerance

**Figure 3:**
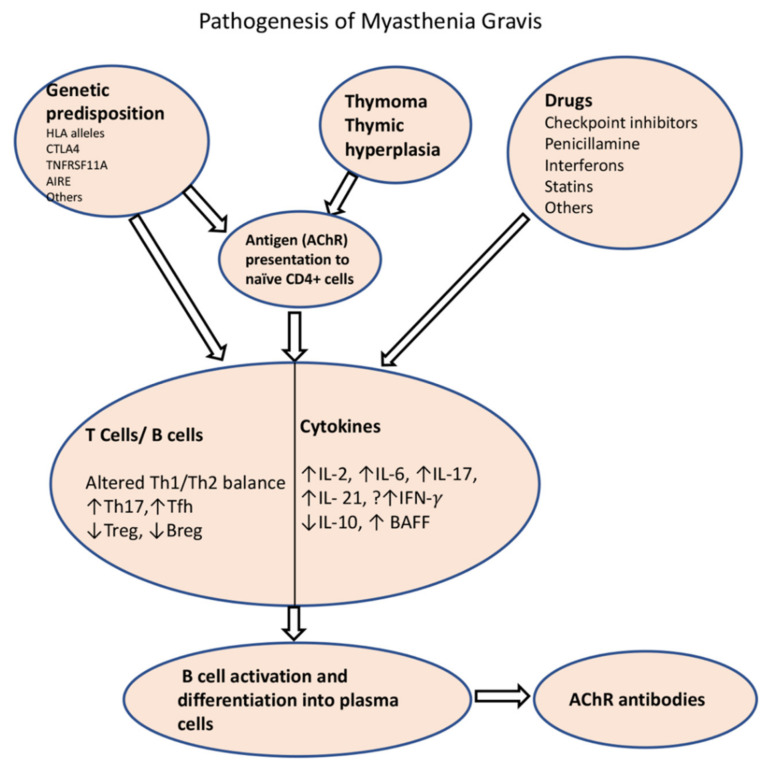
Schematic diagram of pathogenesis of AChR-MG. Impaired tolerance to the AChR is the result of thymoma, thymic dysplasia or due to certain genetic background, which results in presentation of AChR to the naïve T cells by thymic myoid cells or antigen-presenting cells. Among the environmental factors, certain drugs are known to cause de novo MG through alterations of immune homeostasis (drug-induced MG is extensively covered in another paper in this special edition). A number of T cell and B cell subtypes and their cytokines play roles in perturbation of immune homeostasis that results in production of ACR antibodies. HLA: Human Leucocyte antigen; CTLA4: cytotoxic T-lymphocyte-associated protein 4; TNFRSF11A: tumor necrosis factor receptor 4 superfamily, member 11a; AIRE: autoimmune regulator; Th1: T helper 1; Th2: T helper 2; Tfh: T follicular helper; Treg: regulatory T cell; Bregs: regulatory B cells; IL: interleukin; BAFF: B cell activating factor.

B cell tolerance is mediated by clonal deletion or receptor editing in newly generated B cell clones in the bone marrow when they reach the stage of immature B cells. A second checkpoint occurs on the new emigrant/transitional B cells before they enter the mature naïve B cell compartment. Lee et al. found that the frequency of new emigrant/transitional B cells and mature B cells that express polyreactive and autoreactive B cell receptors (BCRs) is higher in both AChR-MG and MuSK-MG, which would support the concept that patients with MG have defects in both central and peripheral B cell tolerance (Figure 3) [100]. As a result, these patients are also at higher risk of developing other autoimmune diseases such as systemic lupus erythematosus, rheumatoid arthritis, and thyroiditis. A break in tolerance is also supported by data from deep sequencing of BCR repertoire showing distinct gene segment usage biases in both V_H_ and V_L_ sequences within the naïve and memory compartments in AChR-MG and MuSK-MG [101].

### 5.3. Role of Thymus in MG

Autoreactive T cells also play an important role in the development of the disease (Figure 3). The T cell selection process takes place in the thymus, mainly in the thymic medulla, where they undergo negative selection for self-antigens [102]. Thymic epithelial cells present self-antigens to developing T cells, either directly or through antigen-presenting cells. If the developing T cells bind strongly to these antigens, they are removed from the repertoire. Mechanisms for removal include (1) clonal deletion, (2) the induction of anergy, or (3) clonal diversion/transformation into regulatory T cells (Tregs). The AChRs, as well as other muscle proteins such as ryanodine and titin, are expressed by thymic myoid and epithelial cells [103]. A key player in T cell autoimmunity is the autoimmune regulator (AIRE) transcription factor, which induces tolerance over autoimmunity, by helping with self-antigen expression in thymic cells. This transcription factor is modulated by estrogen, which may help explain the early female predominance of the disease [104].

MG is uniquely associated with thymus hyperplasia and thymoma. The presence of ectopic germinal centers is associated with early-onset AChR-MG, but not with MuSK-associated MG. The T cell selection process may be impaired in thymic hyperplasia and thymoma. The latter can have defective AIRE expression and can lack thymic medulla, which is involved in the negative selection of T cells, therefore contributing to the release of autoreactive CD4^+^ and CD8^+^ T cells [105]. In addition, the ensuing T cell subset imbalance and cytokine dysregulation leads to the activation of B cells in germinal centers and differentiation into autoreactive B cells and plasma cells [106]. T follicular helper cells (Tfh), which are required for the generation of germinal centers, produce IL-21 and induce immunoglobulin class switching [107]. Microarray analysis in thymic T cells from MG patients revealed a Th1/Th17/Tfh signature [108].

Tfh in lymphoid organs are difficult to assess, so some investigators evaluate the frequency of circulating Tfh (CXC5+ CD4 T cells in peripheral blood) instead. Ashida et al. demonstrated an increase in circulating Tfh with elevated expression of inducible T cell costimulator (ICOS) in a cohort of treatment naïve AChR-MG patients compared to controls [109]. Interestingly, the frequency of circulating Tfh correlated with the severity of MG, and its response to treatment in that study.

#### 5.3.1. Role of T Cells and Cytokines in the Development of MG

Although MG is a B cell-mediated disease, CD4+ T cells and their cytokines contribute to the development of disease. Animal studies showed that mice with depletion of CD4+ T cells or class II major histocompatibility complex (MHC II) did not develop the experimental autoimmune MG after sensitization to AChRs [110,111], further confirming their role. Wu et al. demonstrated that the induction of tolerance to T cell epitopes prevented the development of MG phenotype in a murine model after immunization with AChRs [112].

Patients with MG have autoreactive Th1 and Th2 cells, but their specific role in autoantibody production is not clear. Cytokine measurements in sera by ELISA or by flow cytometry often reveal conflicting results. Th2 cytokines such as IL-4 are known to play a role in the induction of B cells; therefore, it is believed that a humoral Th2 response has a direct role in the immunopathogenesis of the disease [113]. On the other hand, CD4+ T cells with a Th1 profile have also been shown to play a role in MG. For example, patients with MG have high levels of IFN-γ-secreting Th1 cells in the peripheral blood [114]. It is unclear how IFN-γ promotes the production of autoantibodies, but it is believed to induce MHC II and costimulatory molecules in adjacent tissues, such as myocytes, rendering them with the ability to present antigens and promote antibody response. There is some evidence that non-complement fixing isotypes such as anti-MuSK IgG4 are regulated by Th2 cytokines, whereas complement fixing isotypes are regulated by Th1 cytokines [115,116]. Yet, a recent study found an increase in Th2 cytokine IL-4, in addition to IL-10, IL-17 and IL-21 in CD4+ T cells in patients with AChR-MG, when compared to healthy controls [117].

Th17 T cells are associated with tissue-specific autoimmune inflammatory disorders by activating immune cells and promoting their migration into tissues, thus enhancing inflammation overall. These cells are postulated to play a key role in the pathogenesis of MG. Multiple mechanisms are implicated, including the release of interleukin 17 (IL-17), among other cytokines, which indirectly promote immunoglobulin production. Th17 cells also affect the balance of the cytokine profile of Th1 and Th2 cells, thus influencing antibody production [118]. Several studies have shown that patients with MG have elevated levels of Th17 cells and IL-17, which correlate with disease severity and antibody titers [119,120]. In the study by Cao and colleagues, autoreactive T cells from AChR-MG exhibited increased IL-17, IFN-γ, and GM-CSF and diminished IL-10 production [121]. On the other hand, T cell and cytokine profile may change during MG crisis. Huan et al. demonstrated that patients with MG crisis had significantly elevated levels of Th17 as well as Th2-related cytokines IL-4 and IL-13 compared to 6 months post-ventilatory support [122]. Higher frequencies of Th1 and Th17 cytokines were also observed in MuSK-associated MG [123]. During infections, there is likely a perturbation in the cytokine milieu that increases the risk of MG exacerbation.

#### 5.3.2. Regulatory T Cells (Tregs), Regulatory B Cells (Bregs), and B Cell-Activating Factor (BAFF) Signaling in MG

Autoimmune diseases such as MG are precipitated when there is an altered balance between autoreactive T and B cells, and regulatory cell types that suppress them. The latter include Tregs and regulatory B cells, both of which are phenotypically diverse. Tregs suppress the function of other effector T cells and antigen-presenting cells by releasing anti-inflammatory cytokines, such as IL-10 and transforming growth factor beta (TGF-ß), and through the expression of forkhead box protein 3 (FoxP3), among other mechanisms. Th17/Treg imbalance has been reported in patients with MG, especially in those with generalized forms and thymomatous MG [108,124]. Impaired suppressive function of CD4+ Tregs from thymus and peripheral blood cells of MG patients has been demonstrated, though the number of CD4+ Tregs was unchanged in most studies [125,126,127,128].

Regarding Bregs, multiple subsets of B cells with overlapping markers have been reported to produce IL-10 and suppress pro-inflammatory responses, but the establishment of consensus in the field has been hampered by the lack of a unique transcription factor [129]. We and other investigators have reported that MG patients exhibited a decrease in the frequency of CD19^+^CD1d^hi^CD5^+^ and CD19^+^CD24^hi^CD38^hi^ Breg subsets and IL-10-producing B cells within each subset [130,131,132,133]. Taken together, the expansion of Tregs and Bregs or the restoration of its suppressive function is a potential therapeutic strategy in MG.

In contrast to the impaired number and/or function of Tregs and Bregs, there is some evidence of enhanced BAFF signaling in MG. BAFF is a member of the tumor necrosis factor family. BAFF signaling through interaction with BAFF-receptor (BAFF-R) is essential for B cell survival, maturation, and their development into plasmablasts and plasma cells [134]. The levels of circulating BAFF, which is secreted by myeloid cells, are increased in the sera of MG patients [135,136]. In addition, MG patients exhibit an increase in BAFF-R+ B cells [137]. These findings support a role for dysregulated BAFF signaling in MG pathogenesis.

## 6. Conclusions

MG affects all age groups, with peaks in younger women and older men. There is a great variability in the geographical/regional rates of MG, with the incidence and prevalence rates increasing overall, the latter partly due to better awareness and improvements in the diagnosis of the disease. Juvenile MG is more common in people of Asian and African descent. A subset of AChR-MG is caused by a thymoma or thymic hyperplasia. The rest of AChR-MG as well as all of MuSK, Lrp4, and seronegative-MG cases are primarily autoimmune in nature, though influenced by genetic background and environmental factors that are fully understood. Although primarily an antibody-mediated disorder, different T and B cell subsets, including Th2, Th1, Th17, Tfh, Treg and Breg, and their related cytokines play important roles in MG pathogenesis. A deeper understanding of MG subgroups and their distinct immunopathogenic mechanisms will result in the identification of therapeutic targets and the development of targeted treatment strategies.

## Data Availability

No new data were created or analyzed in this study. Data sharing is not applicable to this article.
